# Integrative Analysis Revealed Stemness Features and a Novel Stemness-Related Classification in Colorectal Cancer Patients

**DOI:** 10.3389/fcell.2022.817509

**Published:** 2022-06-03

**Authors:** Meng-Ling Ye, Si-Qi Li, Yi-Xin Yin, Ke-Zhi Li, Ji-Lin Li, Bang-Li Hu

**Affiliations:** Department of Research, Guangxi Medical University Cancer Hospital, Nanning, China

**Keywords:** colorectal cancer, cancer stemness, tumor microenvironment, immunotherapy, prognosis

## Abstract

Cancer stem cells play crucial roles in colorectal cancer (CRC) tumorigenesis and treatment response. This study aimed to determine the value of the mRNA stemness index (mRNAsi) in CRC and introduce a stemness-related classification to predict the outcome of patients. mRNAsi scores and RNA sequence data of CRC patients were analyzed. We found that high mRNAsi scores were related to early-stage CRC and a better patient prognosis. Two stemness-based subtypes (subtype I and II) were identified. Patients in subtype I presented a significantly better prognosis than those in subtype II. Patients in these two subtype groups presented significantly different tumor immunity scores and immune cell infiltration patterns. Genomic variations revealed that patients in subtype I had a lower tumor mutation burden than those in subtype II. A three-gene stemness subtype predictor was established, showing good diagnostic value in discriminating patients in different subtypes. A prognostic signature based on five stemness-related genes was established and validated in two independent cohorts and clinical samples, showing a better predictive performance than other clinical parameters. We concluded that mRNAsi scores were associated with the clinical outcome in CRC patients. The stemness-related classification was a promising prognostic predictor for CRC patients.

## Introduction

Colorectal cancer (CRC) is the third most common cancer and the fourth most frequent cause of cancer-related death worldwide (Araghi et al., 2019). In recent years, the CRC incidence has been steadily rising worldwide, especially in developing countries (Cao et al., 2020). The pathogenesis of CRC is a complex process involving molecular alterations, gene mutations, as well as inflammatory and immune cells. These are crucial factors contributing to CRC development, treatment response, and prognosis. Although significant advancement has been made in the treatment of CRC, the prognosis of patients at later stages remains poor due to tumor metastasis, chemoresistance, and other factors (Galbraith et al., 2021; Zaborowski et al., 2021). Therefore, screening patients at a high potential risk of treatment failure or poor prognosis is critical.

Among the multiple factors involved in CRC pathogenesis, cancer stem cells are essential components of several cancer types (Friedmann-Morvinski and Verma, 2014; Shibue and Weinberg, 2017). Cancer stem cells are those with stem cell–like characteristics that could convert into various malignant cells with different phenotypes, which are more likely to drive tumor formation, growth, and progression, ultimately affecting treatment response, tumor relapse, and prognosis (Reya et al., 2001). Therefore, finding a quantitative indicator to evaluate the degree of oncogenic dedifferentiation might help predict the outcome of cancer patients. In 2018, Malta et al. (2018) introduced the stemness index to assess the degree of oncogenic dedifferentiation through a one-class logistic regression machine learning algorithm to analyze the epigenetic and transcriptomic features derived from non-transformed stem cells and the differentiated progeny, including RNA-based stemness index (mRNAsi), DNA methylation-based stemness index, and epigenetically regulated-mRNAsi (EREG-mRNAsi). They implemented these indices to evaluate the stemness of each tumor sample in The Cancer Genome Atlas (TCGA) database, showing that these indices could precisely predict metastatic events, interpret intratumoral heterogeneity, and assess the prognosis of patients. In particular, by analyzing transcriptomic data of cancer samples, the mRNAsi yields a better prognostic value in reflecting the cancer stemness.

To date, several studies have utilized the stemness score to determine the prognosis, immunotherapy response, and clinical outcomes of various cancers, such as lung cancer (Liao et al., 2020), glioblastoma (Wang et al., 2021a), and cutaneous melanoma (Wang et al., 2020). However, little is known about the role of the stemness score in CRC, although cancer stem cells are the main culprits involved in CRC therapy resistance and disease recurrence (Zeuner et al., 2014; Das et al., 2020). Therefore, in this study, we analyzed the association of the stemness score with CRC and divided patients into two stemness-based subtype groups. Next, we examined the differences in the clinical outcomes, genomic variations, and tumor microenvironment (TME) of patients in the two stemness subtype groups and constructed a subtype predictor and prognostic signature. In our study, we aimed to explore the clinical value of stemness scores in CRC to enable clinicians to provide appropriate treatment to CRC patients on the basis of stemness subtypes.

## Materials and Methods

### Data Collection and Differential Gene Expression Analysis

The CRC RNA sequence and mutation datasets (TCGA-COADREAD), including 570 colon cancer and 192 rectal cancer samples, were downloaded from TCGA database. The corresponding demographic (age, sex, histological type, and TNM stage) and survival data were extracted. The mRNA stemness index (mRNAsi) of each CRC sample from TCGA database was obtained from a previously published study (Malta et al., 2018). The mRNAsi was represented using a value ranging from 0 to 1. A high mRNAsi score indicated high tumor dedifferentiation and cancer stem cell activity. Two gene expression omnibus (GEO) datasets, GSE29621 and GSE39582, were used to validate the predictive value of the prognostic signature. Another three GEO datasets, GSE73360, GSE50421, GSE89076 and GSE62932 were used to verify the expression of stemness-related genes between tumor and normal tissues. The raw data of each dataset were preprocessed using quantile normalization and Log_2_ transformation. The differentially expressed genes (DEGs) between two groups were screened using the “edgeR” package for TCGA dataset and the “limma” package for GEO datasets. An absolute value of a log_2_ fold change (logFC) > 0.5 and a *p*-value < 0.05 were used as the screening criteria for DEG selection.

### Gene Functional Enrichment Analysis

The Gene Ontology (GO) and Kyoto Encyclopedia of Genes and Genomes (KEGG) functional enrichment analyses were conducted to determine the biological function of DEGs, including biological processes (BPs), cellular components (CCs), and molecular functions (MFs). Furthermore, the signaling pathways in which DEGs were enriched were determined using the “clusterProfiler” R package (Wu et al., 2021), with thresholds of *p* < 0.01 and FDR < 0.05. Moreover, Gene set variation analysis (GSVA) was applied to screen significantly enriched pathways between two groups using the Molecular Signatures Database (MSigDB) version 7.4 (Liberzon et al., 2015).

### Tumor Microenvironment Pattern Analysis

The TME contains tumor cells, immune cells, stromal cells, and other cell types closely related to treatment response and patient prognosis. In this study, the Estimation of Stromal and Immune cells in Malignant Tumor tissues using Expression data (ESTIMATE) algorithm (Yoshihara et al., 2013), which generates four types of immune indexes, including immune score, stromal score, ESTIMATE score, and tumor purity, was employed to evaluate the TME in CRC. In addition, tumor-infiltrating immune cells (TIICs) were quantified using the CIBERSORT algorithm (Newman et al., 2015), generating 22 types of TIICs based on gene expression.

### Classification of the Stemness-Related Gene for CRC Patients

The “ConsensusClusterPlus” package (Wilkerson and Hayes, 2010) in R, which provides quantitative stability evidence to determine a cluster count and cluster membership in an unsupervised analysis, was used to perform unsupervised consensus clustering of the samples based on the expression of stemness-related genes. The cumulative distribution function (CDF) curves were used to determine the optimal number of clusters, indexed by k value from 2 to 6. The proportion of the ambiguous clustering algorithm and the consensus Heatmap were also determined using the package.

### Construction of the Diagnostic Predictor and Prognostic Signature

For the diagnostic predictor of different stemness subtypes, CRC patients were randomly classified into training and test groups. The least absolute shrinkage and selection operator (LASSO) regression, extreme gradient boosting (XGBoost), and a logistic regression model were used to screen the most relevant genes by analyzing the expression of stemness-related genes. The performance of the different algorithms was assessed using receiver operating characteristic (ROC) curves, and then the areas under the curve (AUC) were compared between the two groups. The prognostic signature was constructed by multiplying the expression and the coefficients of each gene from the multiple logistic regression models to generate the risk score according to the following formula: risk score = (β mRNA_1_ × expression of mRNA_1_) + (β mRNA_2_ × expression of mRNA_2_) +…+ (β mRNA_n_ × expression of mRNA_n_). The performance of the prognostic signature from TCGA dataset was validated in two independent cohorts.

### RT-PCR Assay Using CRC Clinical Samples

This experiment was approved by the ethics committee of our local hospital. Thirty fresh CRC tissues and corresponding adjacent normal tissues were collected from our hospital between January 2020 and March 2021. Total RNA from CRC tissues was isolated using the TRIzol reagent (Invitrogen; Waltham, MA, United States) according to the manufacturer’s instructions. The gene primers used for RT-PCR are listed in Supplementary Table S1. The RT-PCR procedure was performed using the SYBR Premix Ex Taq kit following the manufacturer’s instructions. The relative gene expression of each gene was calculated using the 2^−ΔΔCT^ method.

### Statistical Analysis

All statistical analyses were conducted using the R software (Version: 3.6.5). The independent Student’s t-test or Mann–Whitney U test for continuous data was used to compare two groups where appropriate. Analysis of variance was used to compare differences among three groups. Kaplan–Meier analysis and the log-rank test were used to compare the overall survival (OS). Correlation analysis was performed using Pearson’s correlation analysis using the “ggstatsplot” package (Patil, 2021). LASSO regression and XGBoost were performed to screen significant genes using the “glmnet” and “XGBoost” packages, respectively. Univariate and multivariate analyses were conducted using the Cox proportional regression model. A two-tailed *p*-value < 0.05 was considered statistically significant.

## Results

### Stemness Scores Associated With Clinical Features in CRC Patients

**FIGURE 1:**
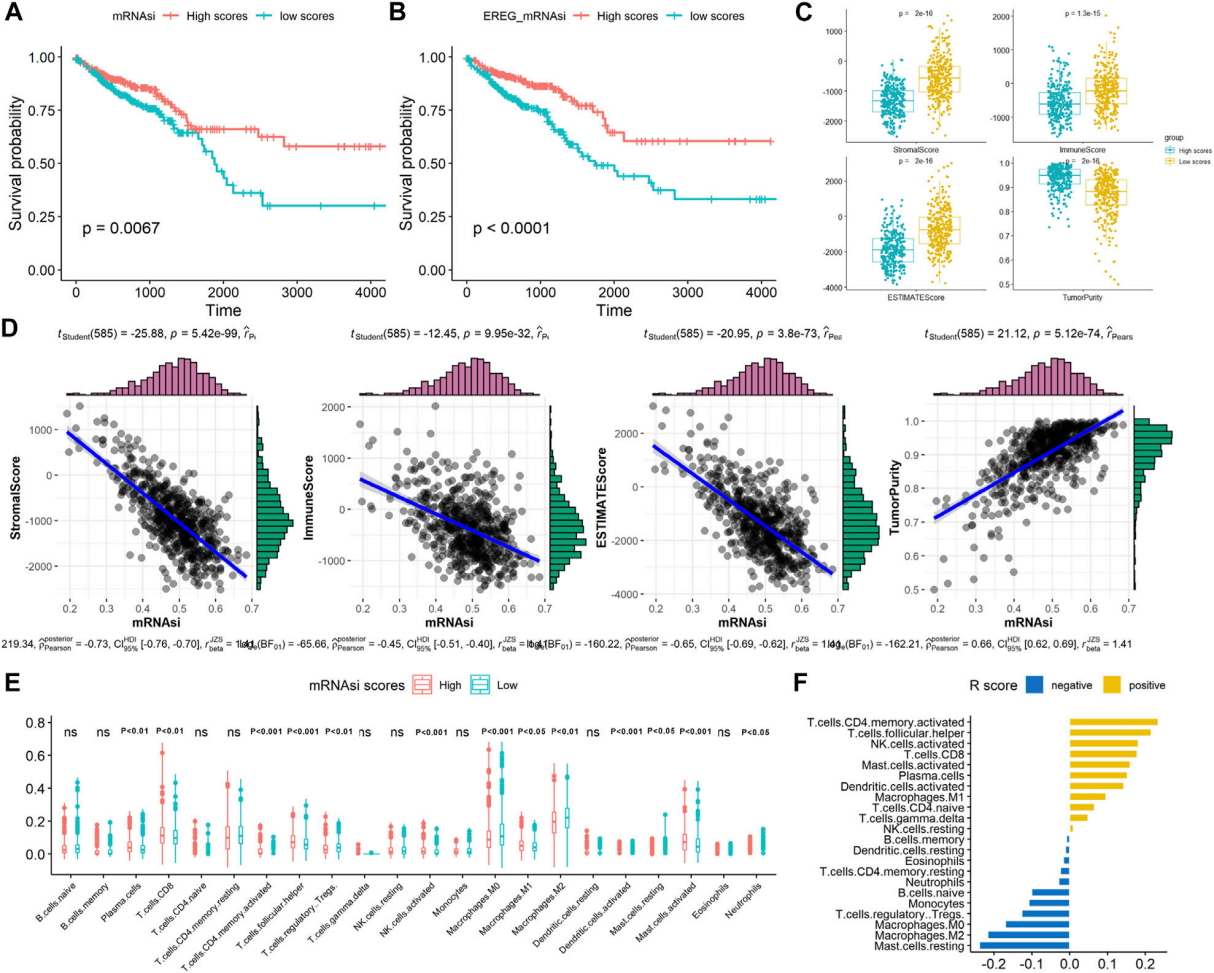
Associations between the stemness scores and survival and TME patterns in CRC. Kaplan–Meier analyses indicated significantly better OS in the high **(A)** mRNAsi scores and **(B)** EREG mRNAsi scores; **(C)** Comparison of immune, stromal, ESTIMATE scores, and tumor purity between high and mRNAsi scores; **(D)** Correlation of mRNAsi scores with immune, stromal, ESTIMATE scores, and tumor purity; **(E)** Comparison of TIICs between high and mRNAsi scores; **(F)** Correlation of mRNAsi scores with TIICs (R value).

The workflow of this study is presented in Supplementary Figure S1. Patients with CRC were divided into two groups, including 293 and 294 patients, respectively, using the median value of the mRNAsi scores, and the association between mRNAsi and clinical features was analyzed. As summarized in Table 1, most patients with high stemness scores were at the early N stage (N0) and clinical stage (I+II) compared with those with low mRNAsi scores (*p* < 0.05), no significant differences were observed between the high and low mRNAsi scores regarding patient age, sex, tumor location, histological type, T stage, and M stage (*p* > 0.05). Kaplan–Meier analysis indicated that patients with high mRNAsi scores or EREG-mRNAsi scores had significantly better OS (mRNAsi: HR = 1.653, log-rank *p* = 0.007; EREG-mRNAsi: HR = 2.099, log-rank *p* < 0.001) than those with low mRNAsi scores (Figures 1A,B).

**TABLE 1:** Comparison of clinical feature between high and low mRNAsi scores.

	High scores (*N* = 293)	Low scores (*N* = 294)	P overall
Age (years)	66.1 ± 12.7	66.5 ± 12.8	0.694
Gender			0.77
Female	135 (46.1%)	140 (47.6%)	
Male	158 (53.9%)	154 (52.4%)	
Location			0.759
Colon	215 (73.4%)	220 (74.8%)	
Rectal	78 (26.6%)	74 (25.2%)	
Histological type			0.152
Adenocarcinoma	258 (88.1%)	246 (83.7%)	
Mucinous	29 (9.90%)	44 (15.0%)	
T stage			0.065
T1	13 (4.44%)	7 (2.38%)	
T2	54 (18.4%)	47 (16.0%)	
T3	202 (68.9%)	198 (67.3%)	
T4	24 (8.19%)	42 (14.3%)	
N stage			0.002
N0	191 (65.2%)	148 (50.3%)	
N1	58 (19.8%)	79 (26.9%)	
N2	43 (14.7%)	66 (22.4%)	
NX	1 (0.34%)	1 (0.34%)	
M stage			0.286
M0	225 (76.8%)	213 (72.4%)	
M1	32 (10.9%)	48 (16.3%)	
MX	33 (11.3%)	30 (10.2%)	
Clinical stage			0.03
I	55 (18.8%)	46 (15.6%)	
II	123 (42.0%)	97 (33.0%)	
III	74 (25.3%)	93 (31.6%)	
IV	31 (10.6%)	50 (17.0%)	
NA	10 (3.41%)	8 (2.72%)	
OS	0.17 (0.37)	0.24 (0.43)	0.033
OS time (days)	886 (825)	754 (620)	0.028

OS: overall survival.

### Associations Between the Stemness Scores and Tumor Microenvironment Patterns

In TME evaluations using the ESTIMATE algorithms, we found that high mRNAsi scores were associated with lower immune and stromal ESTIMATE scores and higher tumor purity than were low mRNAsi scores (Figure 1C, *p* < 0.05). Correlation analysis revealed that mRNAsi scores negatively correlated with the immune, stromal, and ESTIMATE scores but positively correlated with tumor purity in CRC, indicating that the infiltration of immune and stromal cells decreased with the increased of the CRC stemness (Figure 1D). Next, the number of 22 types of TIICs in CRC was calculated using the CIBERSORT algorithm. As shown in Figure 1E, high mRNAsi scores were significantly associated with plasma cells, CD8-positive T cells, T follicular helper cells, activated mast cells, and activated natural killer cells (*p* < 0.001). In contrast, low mRNAsi scores were significantly associated with M0 and M2 macrophages. Correlation analysis revealed that high mRNAsi scores significantly positively correlated with activated memory CD4 T cells and T follicular helper cells, and negatively correlated with resting mast cells and M2 macrophages (*p* < 0.001; Figure 1F). Collectively, these results indicated that high stemness scores were associated with low TME scores in CRC.

### Identifying Differentially Expressed Genes Related to the mRNAsi Scores and Functional Analysis

**FIGURE 2:**
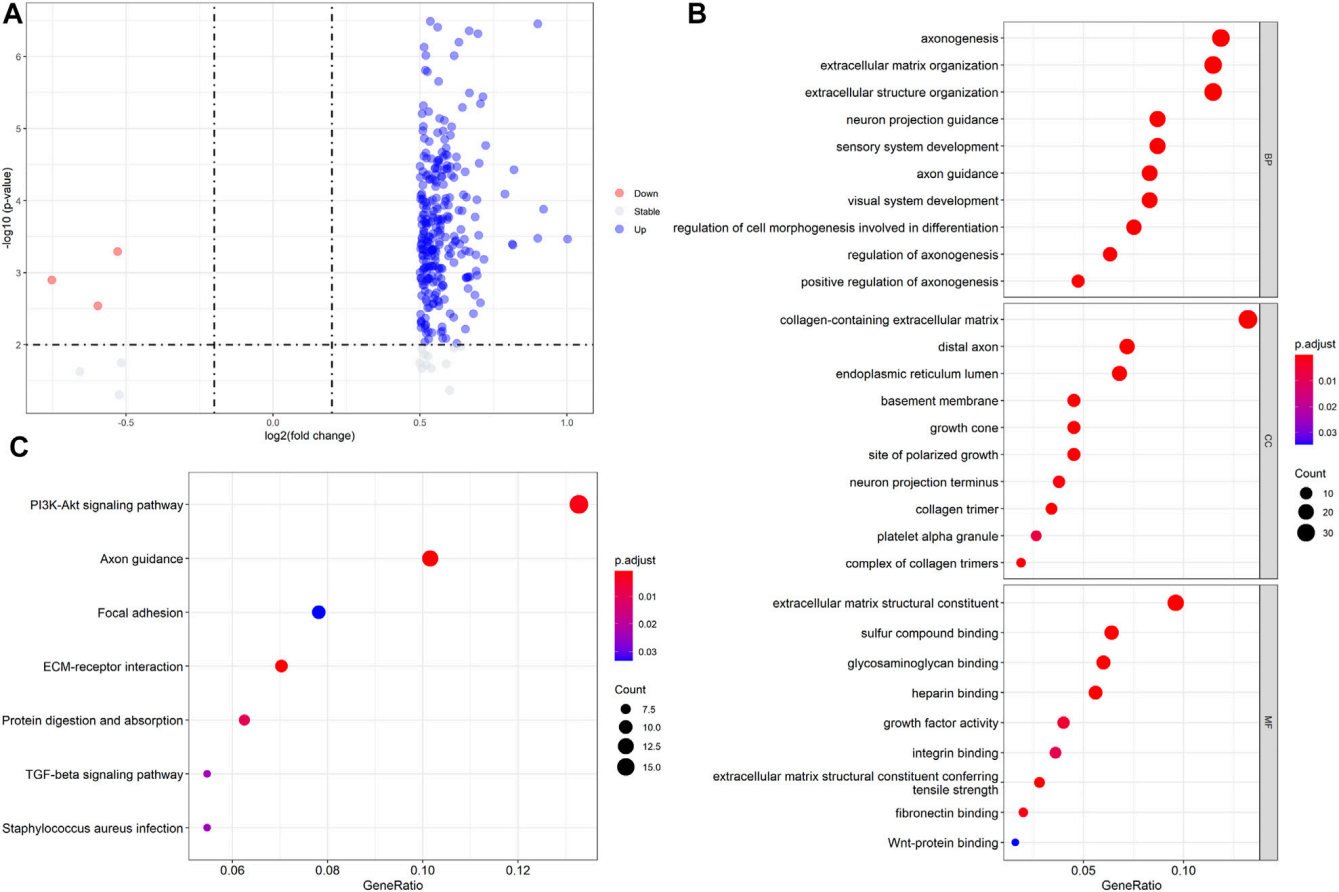
Identifying differentially expressed genes (DEGs) related to mRNAsi scores and the functional analysis. **(A)** Volcano plot of DEGs between high and mRNAsi scores; **(B)** GO functional enrichment analysis for the DEGs; **(C)** KEGG analysis for the DEGs.

Since the different mRNAsi scores in CRC patients had prognostic value, we further utilized the differential expression analysis using the high and low mRNAsi scores. Based on the selected thresholds (logFC > 0.5 and *p* < 0.05), a total of 290 stemness-related DEGs related to mRNAsi scores were identified, including 284 upregulated and six downregulated DEGs (Figure 2A, Supplementary Table S2). Next, functional enrichment analysis of the 290 DEGs was performed using clusterProfiler algorithms. The most enriched BPs were axonogenesis, extracellular matrix organization, and extracellular structure organization; the most enriched CCs were collagen-containing extracellular matrix, distal axon, and endoplasmic reticulum lumen; the most enriched MFs were extracellular matrix constituent, sulfur compound binding, and glycosaminoglycan binding. The significantly enriched KEGG pathways were the PI3K-Akt, axon guidance, and focal adhesion pathways (Figures 2B,C). These results implied that these 290 stemness-related DEGs participated in these processes and pathways in CRC pathogenesis.

### Identification of Two Stemness Subtypes Associated With the Clinical Outcome in CRC Patients

**FIGURE 3:**
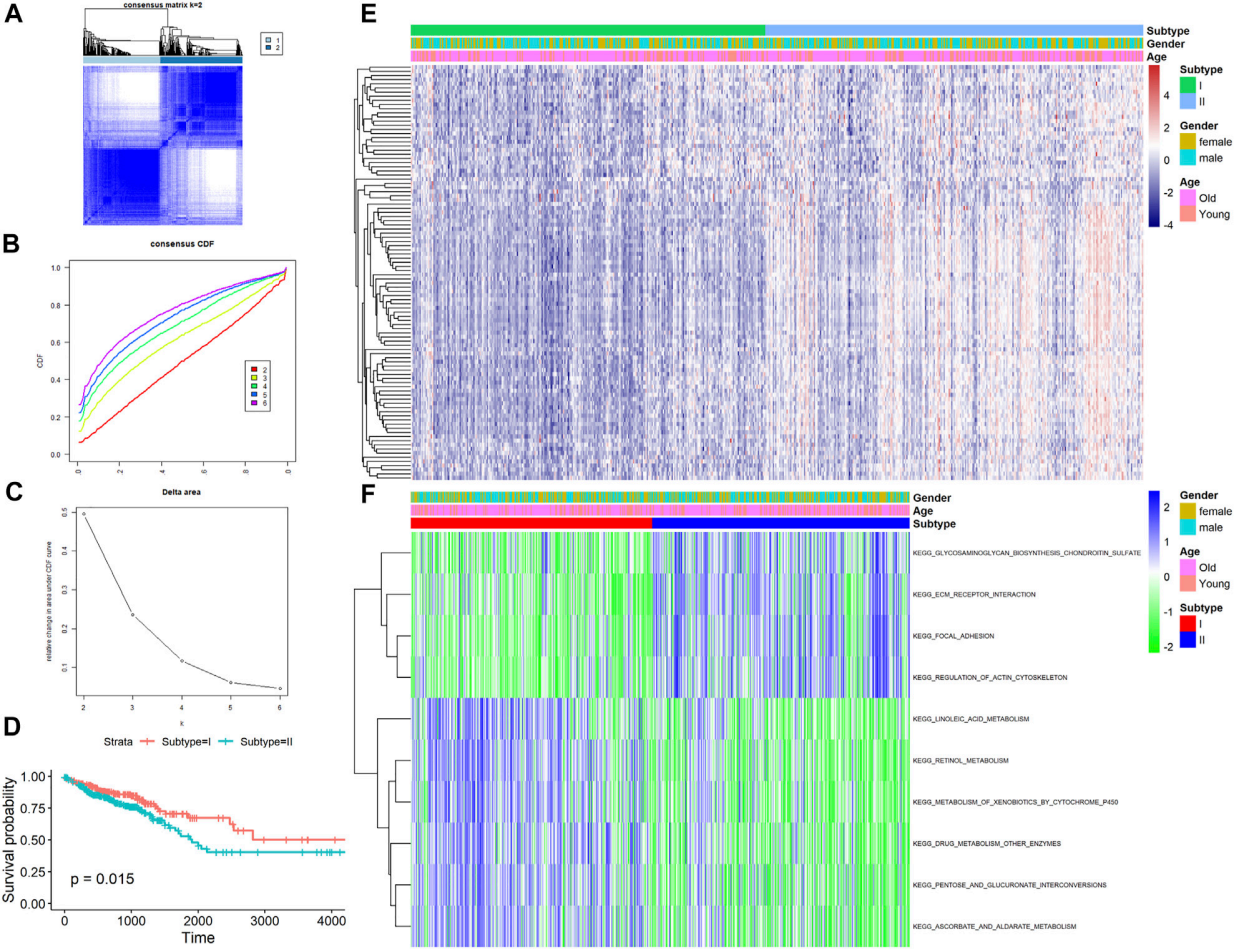
Identification of two stemness subtypes with distinct survival outcomes and functional enrichment. **(A)** Heatmap of the consensus clustering matrix for k = 2; **(B)** CDF curves of the consensus score from k = 2 to 9. **(C)** The relative change in the area under the CDF curve from k = 2 to 9. **(D)** Kaplan–Meier analysis exhibited significantly better OS in stemness subtype I; **(E)** Heatmap of the expression patterns of top 100 DEGs between stemness subtype I and stemness subtype II. **(F)** Heatmap illustrated the enrichment scores of significantly enriched pathways evaluated by GSVA analysis between stemness subtype I and II.

To classify CRC patients into different stemness-based subtypes, we used an unsupervised consensus clustering method by analyzing the expression of 290 stemness-related DEGs based on the differential expression analysis for mRNAsi scores. On the basis of the AUC of the CDF curve, we found that the optimal value of clusters for the cohort was two, implying that the k value was two (Figures 3A–C). Thus, the CRC patients in this cohort were divided into two subtypes, namely, stemness subtype I (284 patients, 48.4%) and stemness subtype II (303 patients, 51.6%).

Then, we explored the association of the two stemness subtypes with patient survival. The mean OS time in stemness subtype I was longer compared with that in stemness subtype II (886 vs. 754 days). Kaplan–Meier analysis also suggested that CRC patients in stemness subtype I have significantly longer OS value than did those in stemness subtype II (HR = 1.571, log-rank *p* = 0.015; Figure 3D).

Next, the molecular pathway and underlying mechanisms involved in the two stemness subtypes were determined using the GSVA algorithm in CRC. As shown in Figures 3A,E total of 185 significantly enriched pathways were identified between stemness subtypes I and II. The most significant pathways in stemness subtype I were the glycosaminoglycan biosynthesis chondroitin sulfate, ECM receptor interaction, and focal adhesion pathways, whereas those in stemness subtype II were the linoleic acid metabolism, retinol metabolism, and metabolism of xenobiotics by cytochrome p450 pathways.

Afterwards, we compared the clinical features of CRC patients between the two stemness subtypes. As summarized in Table 2, a higher number of patients in the stemness subtype I were at the early T stage (T1+T2), N stage (N0), and clinical stage (I+II) than were those in the stemness subtype II (*p* < 0.05). However, no significant difference between the two subtypes regarding patient age, gender, tumor location, histological type and M stage, microsatellite instability (MSI) status, and treatment strategy (*p* > 0.05). To be noted, we compared the stemness-based subtypes to the classification with different mRNAsi scores. We found a significant difference between these two classifications regarding the sample number (*p* < 0.001), with 207 samples overlapping between these two classifications. These results suggested that most patients in the stemness subtype I group were at the early stage of CRC, indicating higher levels of neoplastic stemness and better prognosis.

**TABLE 2:** Comparison of clinical features between subtype I and subtype II.

	Subtype I (*N* = 284)	Subtype II(*N* = 303)	P overall
Age (years)	66.3 ± 12.4	66.4 ± 13.1	0.949
Gender			1.000
Female	133 (46.8%)	142 (46.9%)	
Male	151 (53.2%)	161 (53.1%)	
Location			0.577
Colon	207 (72.9%)	228 (75.2%)	
Rectal	77 (27.1%)	75 (24.8%)	
Histological type			0.053
Adenocarcinoma	250 (88.0%)	254 (83.8%)	
Mucinous	27 (9.51%)	46 (15.2%)	
T stage			<0.001
T1	13 (4.58%)	7 (2.31%)	
T2	66 (23.2%)	35 (11.6%)	
T3	186 (65.5%)	214 (70.6%)	
T4	19 (6.69%)	47 (15.5%)	
N stage			0.033
N0	180 (63.4%)	159 (52.5%)	
N1	60 (21.1%)	77 (25.4%)	
N2	43 (15.1%)	66 (21.8%)	
NX	1 (0.35%)	1 (0.33%)	
M stage			0.454
M0	220 (77.5%)	218 (71.9%)	
M1	33 (11.6%)	47 (15.5%)	
MX	28 (9.86%)	35 (11.6%)	
Clinical stage			0.005
I	66 (23.2%)	35 (11.6%)	
II	103 (36.3%)	117 (38.6%)	
III	74 (26.1%)	93 (30.7%)	
IV	33 (11.6%)	48 (15.8%)	
MSI*			0.394
Yes	4 (1.41%)	7 (2.31%)	
No	45 (15.8%)	58 (19.1%)	
Treatment*			0.136
Ancillary	5 (1.76%)	3 (0.99%)	
Chemotherapy	73 (25.7%)	96 (31.7%)	
Immunotherapy	1 (0.35%)	2 (0.66%)	
Other, specify in notes	5 (1.76%)	9 (2.97%)	
TMT	13 (4.58%)	5 (1.65%)	

MSI: Microsatellite instability; TMT: Targeted Molecular therapy; *empty data was removed.

Previously, an international consortium study (Guinney et al., 2015) using large-scale data to show marked interconnectivity between six independent classification systems coalescing into four consensus molecular subtypes (CMSs) with distinguishing features, including: CMS1 (microsatellite instability immune), hypermutated, microsatellite unstable and strong immune activation; CMS2 (canonical), epithelial, marked WNT and MYC signaling activation; CMS3 (metabolic), epithelial and evident metabolic dysregulation; and CMS4 (mesenchymal), prominent transforming growth factor-β activation, stromal invasion and angiogenesis. We compared the previous classification with our classification, and found that the distributions of the molecular subtypes have little difference between subtype I and subtype II (Chi-square test, *p* > 0.05), suggesting that our classification is similar to their classification (Supplementary File Figure S2).

### Stemness Subtypes Possessed Different Tumor Mutation Burdens

**FIGURE 4:**
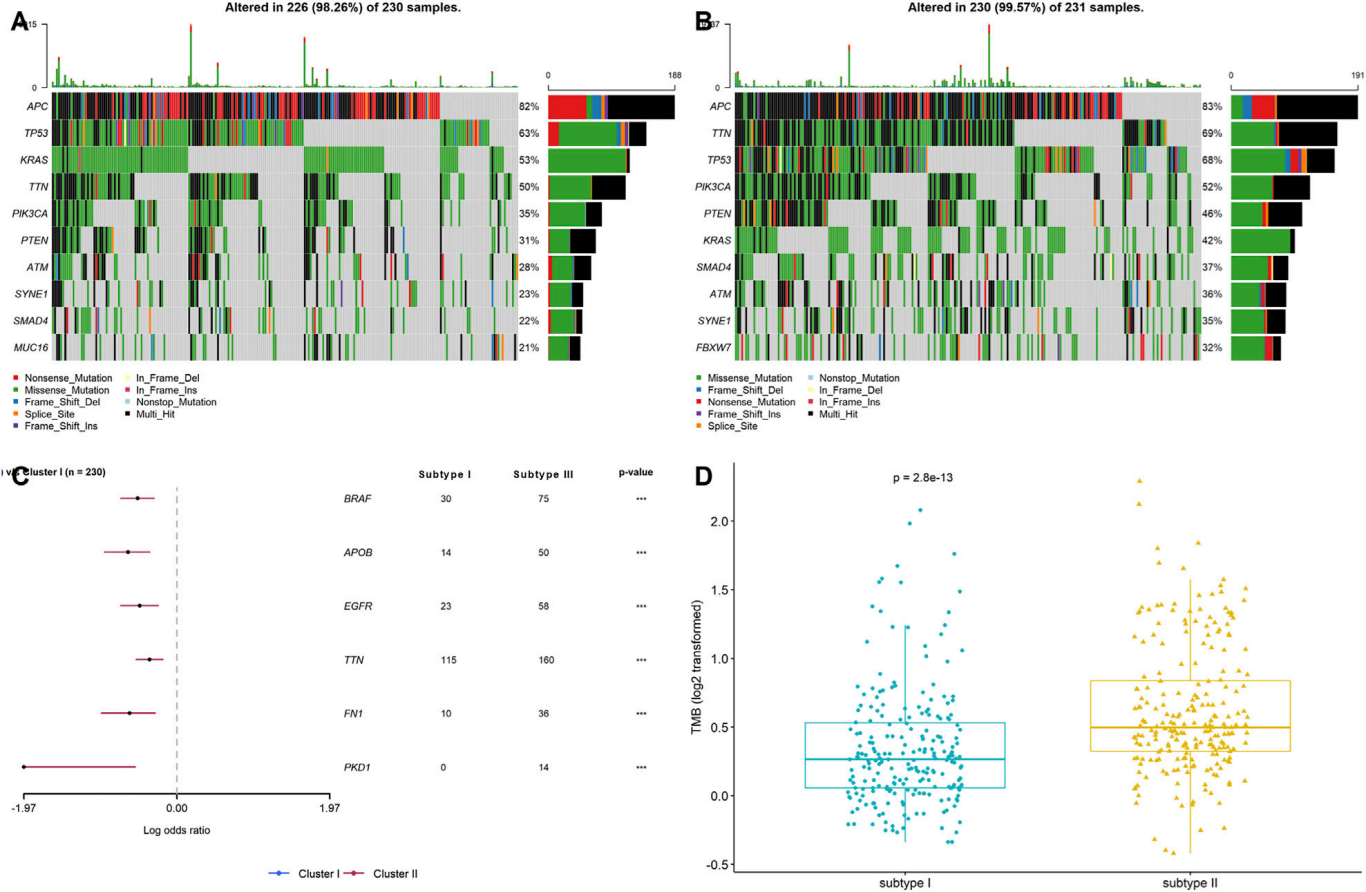
Stemness subtypes possessed different TMB. Waterfall plots showed the top 10 mutated in **(A)** stemness subtype I and **(B)** stemness subtype II; **(C)** Forest plot exhibited the significant mutated genes between the two stemness subtypes; **(D)** The comparisons of TMB between stemness subtype I and stemness subtype II.

Genomic alterations have been reported to modulate the TME in various cancers, including the tumor immunity and immune infiltration patterns in previous studies (Huang et al., 2021; Liu et al., 2021). Hence, gene mutation analysis for the stemness-related DEGs was employed to examine the distinct genomic variation patterns in the two stemness subtypes. As shown in Figures 4A,B, among the top 10 mutated genes of the two stemness subtypes, APC, TTN, TP53, PIK3CA, PTEN, KRAS, SMAD4, ATM, and SYNE1 harbored the highest number of mutations in both subtypes, suggesting that these two subtypes have similar top mutated genes. These mutated genes are known to be involved in CRC pathogenesis. Next, by comparing the gene mutation frequency in stemness subtype I and subtype II, we found that BRAF, APOB, EGFR, TTN, FN1, and PKD1 had a higher mutation frequency in stemness subtype I than in stemness subtype II. However, there was no significant difference regarding the mutation status of APC, TTN, TP53, PIK3CA, PTEN, and KRAS between the two subtypes (Figure 4C). In addition, the results showed that patients in stemness subtype II had substantially higher TMB values than those in stemness subtype I (*p* < 0.001; Figure 4D). These findings suggested significantly different TMB, but the top high-frequency mutated genes showed little difference between the two stemness subtypes.

### Stemness Subtypes had Distinct Tumor Microenvironment Patterns in CRC

**FIGURE 5:**
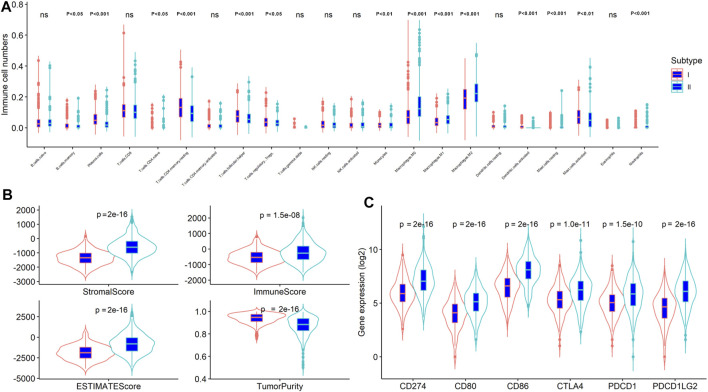
The stemness subtypes had distinct TME patterns in CRC. Comparison of **(A)** TIICs; **(B)** immune, stromal, ESTIMATE scores and tumor purity; **(C)** Immune checkpoint inhibitors between stemness subtype I and stemness subtype II.

On the basic of ESTIMATE algorithm results, the value immune, stromal, and ESTIMATE scores in stemness subtype I were significantly lower than stemness subtype II, while the tumor purity was expectedly higher in stemness subtype I than in stemness subtype II (*p* < 0.001; Figure 5B), suggesting a relatively low abundance of immune cells and stromal cells in the tumors of stemness subtype I. The number of TIICs was quantified using the CIBERSORT algorithm and then compared between stemness subtype I and II. As shown in Figure 5A, the number of plasma cells, resting CD4 memory T cells, T follicular helper cells, monocytes, and activated dendritic cells was significantly higher in stemness subtype I than in stemness subtype II (*p* < 0.001). Furthermore, the number of M0, M1, and M2 macrophages, resting mast cells, and neutrophils was remarkably high in stemness subtype II than in stemness subtype I (*p* < 0.001). Next, we explored the association of stemness subtypes with the six markers of immune checkpoint inhibitors, including PD1 (PDCD1), PD-L1 (CD274), PD-L2 (PDCD1LG2), CTLA4, CD80, and CD86. As shown in Figure 5C, the expression of these markers was expectedly increased in stemness subtype II compared to that in stemness subtype I. These results revealed that tumors in stemness subtype II presented high immune cell levels and were more likely associated with a low immunotherapy response. However, these results were obtained from dataset analysis; thus, this hypothesis should be validated by an independent cohort using proper methods, such as flow cytometry or the RNA sequence.

### Construction of Predictor for the Stemness Subtypes

**FIGURE 6:**
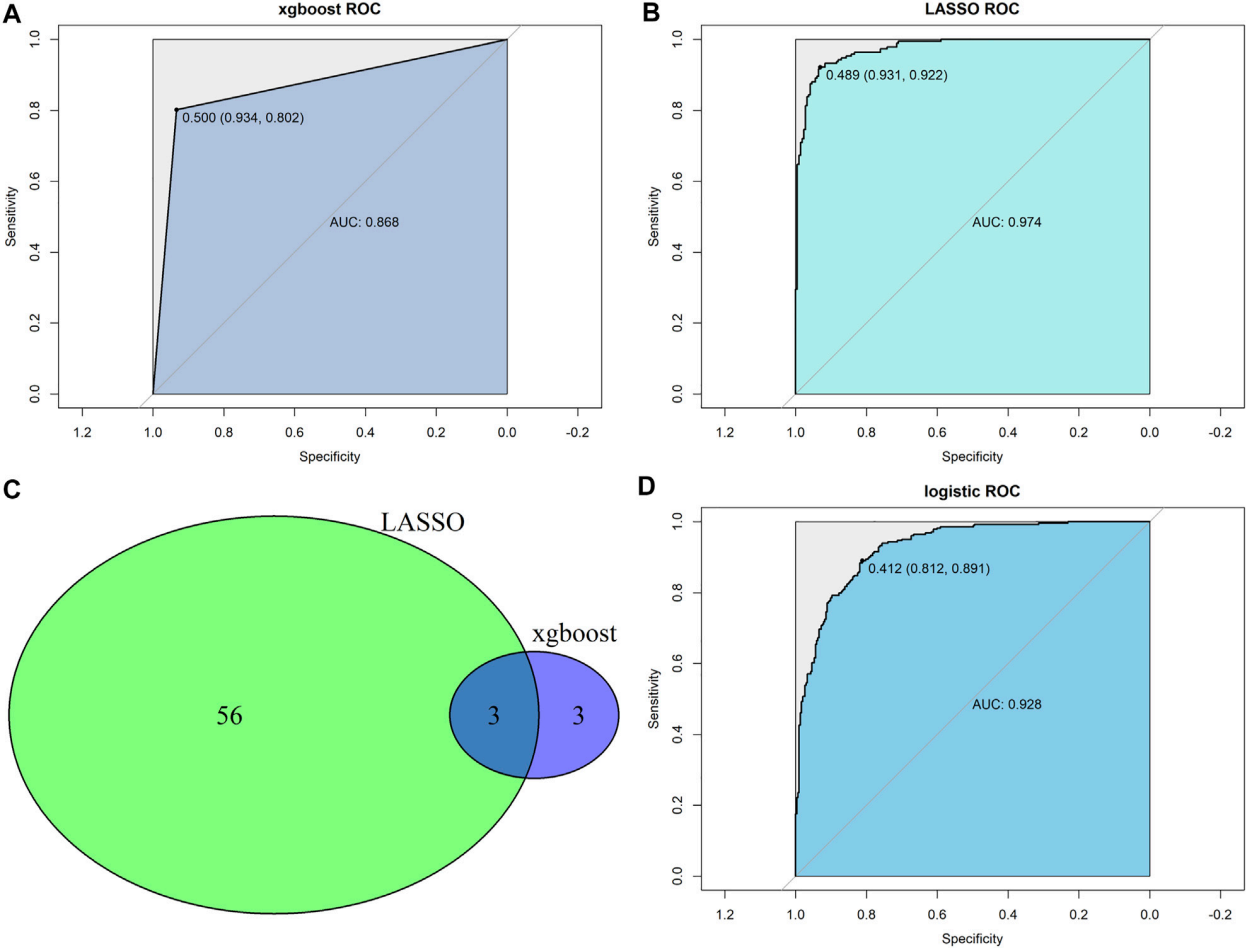
Construction of predictor for the stemness subtypes. **(A)** XGBoost algorithms identified a model predicting the stemness subtypes; **(B)** LASSO algorithms identified a model predicting the stemness subtypes. **(C)** Venn diagram identified overlapped genes between xgboost and LASSO algorithms. **(D)** Multivariate logistic regression model revealed the diagnostic value in predicting the stemness subtypes.

The CRC samples were divided into two sets at a 7:3 ratio, with 410 and 177 patients in the training and testing sets, respectively. Two machine learning algorithms (LASSO and XGBoost) were applied to analyze 290 stemness-related DEGs and identified the key genes associated with the stemness subtypes. The ROC curve revealed that both LASSO and XGBoost results yielded good performances in differentiating stemness subtypes I and II (both AUC values over 0.85), with the AUC values of LASSO being higher than that of the XGBoost algorithm (Figures 6A,B). The LASSO and XGBoost algorithms identified 59 and six genes, respectively. The Venn diagram showed that three genes (GAS1, CHIT1, and COL10A1) overlapped between the two machine learning algorithms results (Figure 6C). Afterward, we conducted a multivariate logistic regression analysis to construct a diagnostic model by incorporating these three genes. As the results suggested, the optimal cutoff value of the model for discrimination was 0.412, implying that patients with scores < 0.412 were grouped in stemness subtype I and stemness subtype II if otherwise. ROC curve analysis demonstrated that the diagnostic, predictive model had an AUC of 0.928 in discriminating the two subtypes, with the sensitivity and specificity as 81.2% and 89.1% in the entire dataset (Figure 6D). In addition, the diagnostic model also had an excellent performance in distinguishing the two stemness subtypes in the test set, with sensitivity, specificity, and AUC of 0.837, 0.918, and 0.941, respectively (Supplementary Table S3).

### Construction and Validation of the CRC Predictor Based on Stemness-Related Genes

**FIGURE 7:**
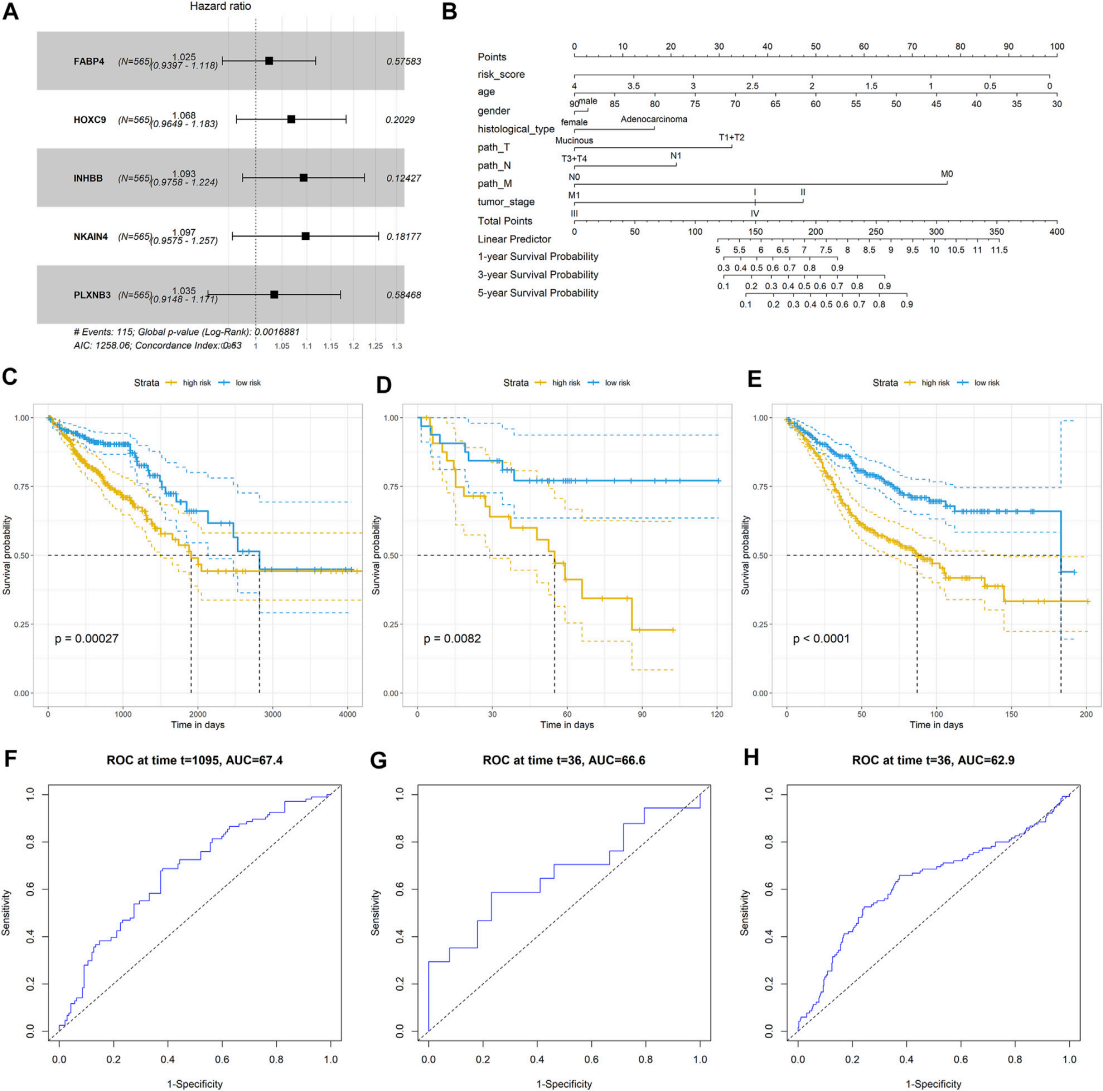
Construction and validation of CRC predictor based on the stemness-related genes. **(A)** Forest plot showed that five stemness- related genes associated with the prognosis of patients; **(B)** Nomogram plot of showed the prognostic value of the gene signature and other clinical features in CRC; Kaplan–Meier analysis indicated the survival rated of patients with high or low risk scores in **(C)** TCGA-COADREAD dataset; **(D)** GSE29621 dataset **(E)** GSE39582 dataset; Prognostic value of the gene signature in **(F)** TCGA-COADREAD dataset; **(G)** GSE29621 dataset; **(H)** GSE36582 dataset.

We further established a prognostic signature based on stemness-related genes to screen the CRC patients with different treatment failure risks or poor prognoses. A total of 290 stemness-related DEGs were incorporated into the univariate Cox regression model and LASSO analysis, and 55 and 11 genes were identified by the two algorithms, respectively. Of note, five genes (FABP4, HOXC9, INHBB, NKAIN4, and PLXNB3) overlapped between these two models (Figure 7A). Thus, we incorporated these five genes into a multivariate Cox regression model and constructed a prognostic signature. The nomogram showed that the signature had a better prognostic value in CRC compared to that of other clinical parameters (Figure 7B). We categorized the CRC patients into two groups according to the median risk score value of the signature. The Kaplan–Meier analysis indicated that CRC patients with a high risk score had a poorer prognosis than those with a low risk score (log-rank *p* < 0.001; Figure 7C). Next, two independent cohorts, GSE29621 (65 samples) and GSE39582 (585 samples) were employed to validate the prognostic value of the signature. Similar to the results from TCGA-COADREAD dataset, the Kaplan–Meier analysis also revealed that CRC patients in the high risk score group had a poorer prognosis than those in the low risk score group (both log-rank *p* < 0.001; Figures 7D,E). Considering that the survival time of the three datasets varied considerably, to guarantee comparable results, we selected 3-year survival time to compare the predictive value. As shown in Figures 7F–H, the ROC values of the three datasets for 3-year survival were similar, all exceeding 60%, suggesting a moderate prognostic value. Taken together, these results suggested that this stemness-related gene signature has better performance in predicting the prognosis of CRC patients than other clinical parameters.

### Validation of the Five Stemness-Related Genes in Independent Cohorts and CRC Clinical Samples

**FIGURE 8:**
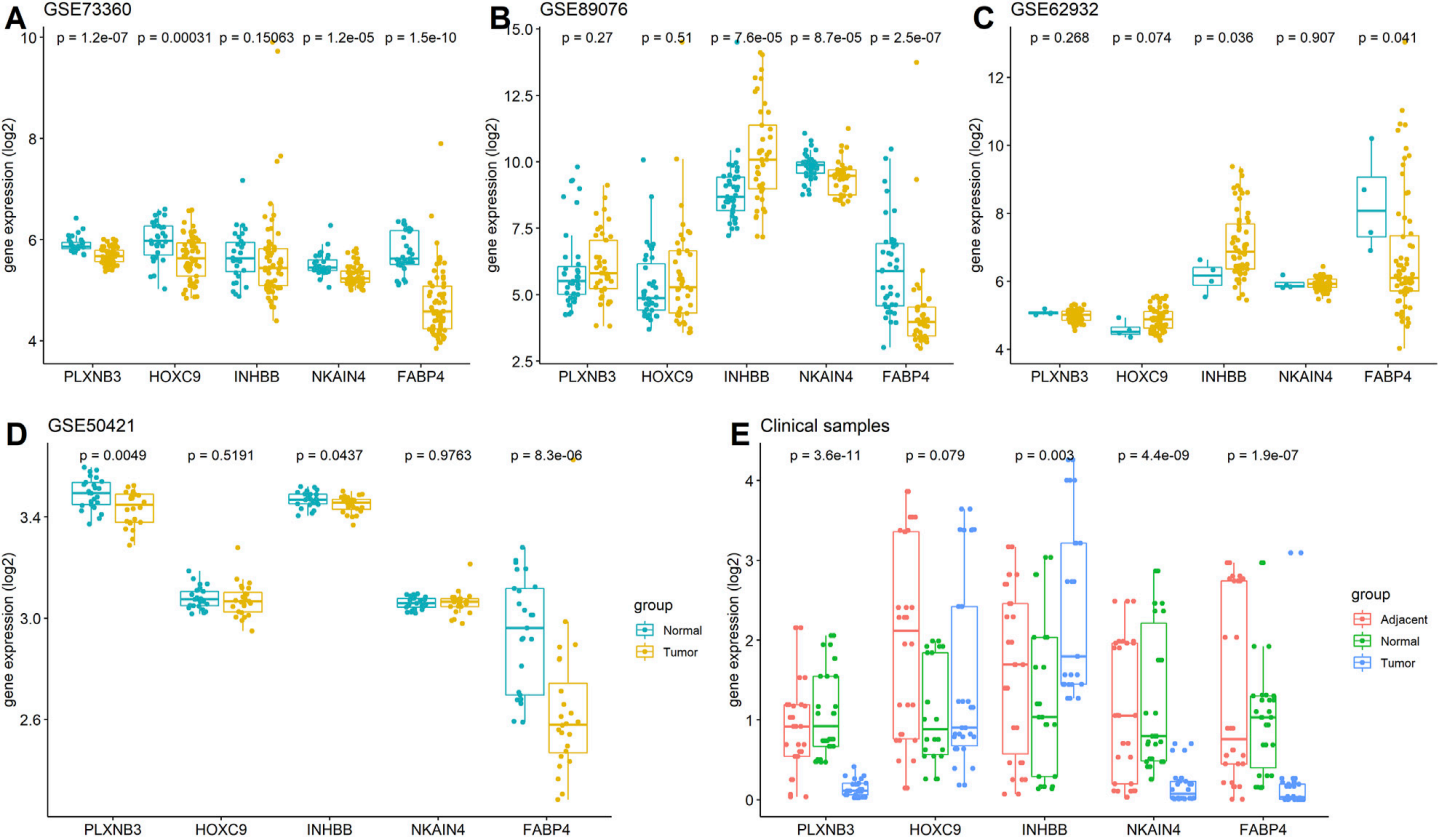
Expressions of FABP4, HOXC9, INHBB, NKAIN4, and PLXNB3 in CRC. **(A)** GSE73360 dataset; **(B)** GSE89076 dataset; **(C)** GSE62932 dataset; **(D)** GSE50421 dataset; **(E)** Clinical tissue tested by RT-PCR assay.

The expression of the five stemness-related genes related to the prognosis of CRC patients was validated in three GEO datasets: GSE73360 (92 samples), GSE50421 (49 samples), GSE89076 (80 samples), and GSE62932 (68 samples) containing normal control and tumor samples. Furthermore, 30 clinical CRC tissues (including tumor tissues and the corresponding adjacent tumor tissues as well as normal tissues) were collected, and the expression of FABP4, HOXC9, INHBB, NKAIN4, and PLXNB3 was determined using RT-PCR. The results showed that the expression of these genes was reduced in tumor tissues compared to that in normal tissues. As listed in Figure 8, low expression of FABP4 in tumor tissues (*p* < 0.05) was validated in five datasets, the lower expression of PLXNB3, INHBB and NKAIN4 in tumor tissues was verified in three datasets (*p* < 0.05), and the decreased expression of HOXC9 and was validated in one dataset, respectively (*p* < 0.05).

## Discussion

Currently, CRC remains an incurable disease because 50% of CRC patients experience tumor relapse and metastasis even if the tumor is removed prior to tumor metastasis (Conti and Thomas, 2011; Jain et al., 2011). Cancer stem cells have intrinsic chemoresistant properties, ultimately leading to chemotherapy failure and cancer recurrence (Zahra et al., 2021). Hence, targeting cancer stem cells or related genes might be a promising strategy for treating CRC. Previous studies indicated that molecular subtypes based on the stemness scores predict outcome in several cancers (Hong et al., 2021; Tan et al., 2021). These subtypes are reportedly associated with the TME or immune therapy in these cancers. Regarding CRC, a recent study reported that the mRNAsi score was negatively related to pathological features but positively associated with OS and recurrence-free survival in CRC patients (Wang et al., 2021b). However, this study did not conduct an in-depth analysis of the association of mRNAsi scores with the clinical outcome and TME and lacked external cohorts to validate the robustness of the results. Therefore, it is necessary to perform a detailed analysis to reveal the clinical value of stemness in CRC.

Here, we conducted a comprehensive analysis of the association of stemness scores with the clinical outcome in CRC patients, showing that the stemness scores were significantly associated with the clinical stage and survival of CRC patients. We explored the function of stemness-related genes and their association with TME. Furthermore, we identified two stemness subtypes based on the stemness scores and determined the pathways involved in the two subtypes and the association of stemness subtypes with the clinical features, survival time, gene mutation, TME, and prognostic value. Moreover, we constructed a predictor for the stemness subtypes and a prognostic signature based on stemness-related genes using machine learning algorithms. The results revealed that the stemness subtypes were significantly associated with the clinical features, TME, TMB, immunotherapy response, and prognostic value. Finally, to increase the reliability of the results, several independent cohorts and clinical samples were used to validate the diagnostic value of the predictor for stemness subtypes and the prognostic value of the prognostic signature in CRC.

At present, immunotherapy has been shown to be a promising option for CRC patients, especially for those unable to undergo radical resection or with tumor metastasis. However, many factors affect CRC immunotherapy, among which cancer stem cells are a key factor that might result in immunotherapy failure. Although the association of cancer stem cells with the immune system has not been well investigated because of experimental limitations (Donini et al., 2021), evidence demonstrates that cancer stem cells have a modulatory effect on the immune system in CRC patients (Xu et al., 2018). In this study, we showed that the stemness scores were significantly associated with the TME, including the immune, stromal scores, and tumor purity, and that they greatly correlated with some TIICs. In addition, the stemness subtypes can clearly discriminate CRC patients with different TME and TMB. Furthermore, we found remarkable associations of stemness subtypes with immune checkpoint inhibitors. All these results strongly suggest that targeting the stemness scores of CRC could effectively identify patients who will benefit from immunotherapy. Interestingly, we found that the TMB differed between the two subtypes, which shared most of the top mutated genes, suggesting that more gene mutations need to be explored in these two stemness-based subtypes.

Since the stemness scores are calculated based on the gene expression profile, which cannot be easily used in the clinical setting, we established a stemness subtype predictor and prognostic signature based on stemness-related genes. The stemness subtype predictor established using three stemness-based genes helps to discriminate the patients of stemness subtype I from those of stemness subtype II. The prognostic signature constructed based on five genes further facilitates the screening of CRC patients at different risk levels in terms of survival. To be noted, regarding the genes used to construct the stemness subtype predictor and prognostic signature, GAS1 (Li et al., 2016a), CHIT1 (Li et al., 2016b), COL10A1 (Patra et al., 2021), FABP4 (Wang et al., 2019), HOXC9 (Hu et al., 2019), INHBB (Yuan et al., 2020), and NKAIN4 (Jin et al., 2020) have been previously found to be associated with CRC development. In contrast, the role of PLXNB3 in CRC has not been yet investigated, although it was found to be overexpressed in breast cancer (Valladares et al., 2006). This evidence suggested that each stemness-related gene is crucial to CRC development and prognosis.

In this study, we showed that patients with stemness subtype I have a better prognosis compared with those with stemness subtype II, and the TMB in stemness subtype I was lower than that in stemness subtype II, which was contrary to the current understanding, implying that high TMB may be a good prognostic factor for immune checkpoint inhibitors. We speculated that at least two reasons might explain these opposite results. First, as shown in Table 2, most of the patients in stemness subtype I were at an early stage of CRC; therefore, their prognosis was better than those in stemness subtype II. Although high TMB was observed in stemness subtype II, the association of TMB with the immunotherapy effect, especially the PD1 treatment, for CRC remains inconsistent in current reports. In addition, as the results showed, the mutation frequency of the most common mutated genes was similar in both stemness subtypes. Second, MSI was considered a more reliable indicator for the immunotherapy effect for CRC patients; however, our study failed to show a significant association of stemness subtypes with the MSI status; thus, the stemness subtypes might not be a good indicator to predict which CRC patients should undergo immunotherapy.

Furthermore, patients in the different subtype groups showed significantly different prognoses. Notably, the stemness subtype predictor and prognostic signature were validated using external independent cohorts and clinical samples, which confirmed the robustness of our results. However, this study also has some limitations. First, this study only used 30 paired clinical samples to validate the results, which undermined the robustness of the conclusion. Therefore, a larger clinical cohort to further confirm the predictive value of the signature is necessary. Second, the association between stemness subtypes and immunotherapy response needs to be verified in patients who had undergone immunotherapy. Third, in the validated analysis, we found that not all gene expression was consistent with previous results; we speculated that some factors, such as different clinical characteristics, sample size, and the heterogeneity of TME might explain the inconsistent results. Therefore, additional experiments, such as flow cytometry or RNA sequencing with a larger sample size, are warranted to comprehensively analyze the TME and validate our results. Fourth, the EREG-mRNAsi is another indicator that reflects the degree of oncogenic dedifferentiation, which was generated using a set of epigenetic regulatory genes associated with stemness. The EREG-mRNAsi reflecting epigenetically regulated mRNAsi and was considered to be complementary to mRNAsi. In our study, we mainly interesting in the mRNA level, in which using mRNAsi is more appropriate, thus we used mRNAsi in our analysis. But EREG-mRNAsi is important indictor to the prognosis of patients, the similar analysis for the CRC patients by using EREG-mRNAsi should be further conduct in the future study by analyzing the epigenetically gene level and compared with the mRNA level. Therefore, our results should be accepted with caution, and studies to address these concerns should be undertaken in the future.

## Conclusion

This study demonstrated that mRNAsi scores are closely related to the TME and survival of CRC patients. The stemness-related classification based on the mRNAsi score represents a promising predictor of the prognosis of CRC patients.

## Data Availability

The original contributions presented in the study are included in the article/Supplementary Material, further inquiries can be directed to the corresponding authors.
