# Supplementary material for: Integrative Analysis Revealed Stemness Features and a Novel Stemness-Related Classification in Colorectal Cancer Patients
Source: Front Cell Dev Biol. 2022 Jun 3;10:817509. doi: 10.3389/fcell.2022.817509 (PMC9204093; doi:10.3389/fcell.2022.817509)
Supplement: Supplementary file 1 [file DataSheet1.doc]

**Supplementary files**


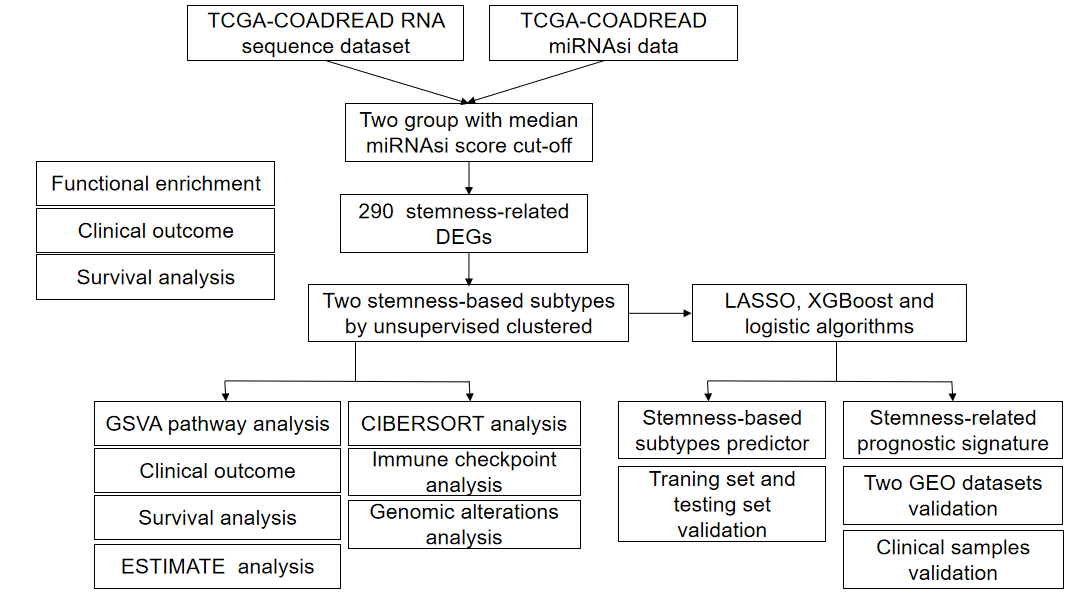


Figure S1 Overall workflow of the study

Table S1 Primer Sequence of genes

| Genes |  | Primer Sequence |
| --- | --- | --- |
| FABP4 | FORWARD | GAGAGGATGATAAACTGGTGGTG |
|  | REVERSE | GCGAACTTCAGTCCAGGTCAA |
| HOXC9 | FORWARD | TCTCACGACAATGAAGACCTCCTA |
|  | REVERSE | CTACAGTCCGGCACCAAACC |
| INHBB | FORWARD | CAGGTCATCCTTGCCAGTTGTAT |
|  | REVERSE | CATTTCCTCCTGCGGTCTCA |
| NKAIN4 | FORWARD | CCAAACTCGTGCCAGAAGAAC |
|  | REVERSE | GAGCCTTTGGGACTGTTTGC |
| PLXNB3 | FORWARD | ACCTGACCCTATCGGACGAA |
|  | REVERSE | ATCTGGGACCTTGTAGTGTTGC |

Table S2 Identifying differentially expressed genes between high and low mRNAsi score

| Gene | logFC | P.value | Gene | logFC | P.value | Gene | logFC | P.value |
| --- | --- | --- | --- | --- | --- | --- | --- | --- |
| TEKT3 | 0.54 | 0 | EBF3 | 0.56 | 0 | MYH10 | 0.5 | 0 |
| NEFL | 0.9 | 0 | SPTBN5 | 0.56 | 0 | TCL1A | 0.69 | 0 |
| ADAMTSL4-AS2 | 0.56 | 0 | RGL3 | 0.7 | 0 | ZFPM2 | 0.54 | 0 |
| TSPAN32 | 0.67 | 0 | EGR2 | 0.56 | 0 | MROH3P | 0.51 | 0 |
| CLDN11 | 0.7 | 0 | ABCA6 | 0.59 | 0 | MATN3 | 0.59 | 0 |
| CORO2B | 0.63 | 0 | PMEPA1 | 0.5 | 0 | PRICKLE1 | 0.53 | 0 |
| OBSCN-AS1 | 0.51 | 0 | CACNB4 | 0.6 | 0 | SOX6 | 0.52 | 0 |
| FYN | 0.52 | 0 | ENTPD3 | 0.63 | 0 | CCDC80 | 0.6 | 0 |
| FST | 0.62 | 0 | ARSI | 0.55 | 0 | FBLN2 | 0.58 | 0 |
| CDYL2 | 0.52 | 0 | RSPO1 | 0.56 | 0 | DBH | 0.51 | 0 |
| IQCN | 0.53 | 0 | TENM4 | 0.59 | 0 | TMEM132E | 0.52 | 0 |
| DRAXIN | 0.56 | 0 | DPYSL3 | 0.55 | 0 | GALNT15 | 0.56 | 0 |
| CLIC6 | 0.67 | 0 | TSHZ2 | 0.59 | 0 | DPYSL4 | 0.52 | 0 |
| C3 | 0.71 | 0 | TBX18 | 0.82 | 0 | BNC2 | 0.56 | 0 |
| HAS1 | 0.71 | 0 | BOC | 0.58 | 0 | C5orf38 | 0.92 | 0 |
| ADAMTSL4 | 0.51 | 0 | PNMA8B | 0.51 | 0 | FLJ16779 | 0.63 | 0 |
| BEAN1 | 0.64 | 0 | PLXNB3 | 0.65 | 0 | GAP43 | 0.64 | 0 |
| GREB1 | 0.53 | 0 | FBLN1 | 0.55 | 0 | CECR7 | 0.55 | 0 |
| MIR29B2CHG | 0.51 | 0 | DACT1 | 0.53 | 0 | SNCA | 0.51 | 0 |
| NPAS3 | 0.57 | 0 | COL22A1 | 0.61 | 0 | SMARCA1 | 0.51 | 0 |
| SDK1 | 0.58 | 0 | ABCA10 | 0.52 | 0 | TMEM130 | 0.54 | 0 |
| FAT2 | 0.51 | 0 | TP63 | 0.57 | 0 | CD300E | 0.59 | 0 |
| HSPA7 | 0.61 | 0 | SLC4A3 | 0.58 | 0 | BMPR1B | 0.69 | 0 |
| SLC22A20P | 0.51 | 0 | HS3ST3A1 | 0.5 | 0 | APCDD1L | 0.6 | 0 |
| LY6H | 0.58 | 0 | TUBB2B | 0.62 | 0 | PALM2AKAP2 | 0.55 | 0 |
| AVPR2 | 0.56 | 0 | CCDC8 | 0.54 | 0 | PDE11A | 0.56 | 0 |
| EPHB6 | 0.6 | 0 | WHAMMP2 | 0.53 | 0 | KANK4 | 0.58 | 0 |
| AGPAT4 | 0.52 | 0 | KLF12 | 0.53 | 0 | TPTEP1 | 0.51 | 0 |
| NKAIN4 | 0.58 | 0 | SEZ6L | 0.55 | 0 | GPAT2 | 0.52 | 0 |
| COL16A1 | 0.53 | 0 | C11orf21 | 0.56 | 0 | GFRA1 | 0.57 | 0 |
| SLIT2 | 0.72 | 0 | NTRK3 | 0.58 | 0 | SV2B | 0.59 | 0 |
| GPIHBP1 | 0.59 | 0 | WHAMMP3 | 0.51 | 0 | EMBP1 | 0.57 | 0 |
| GRIK3 | 0.58 | 0 | LAMA2 | 0.55 | 0 | ABCA9 | 0.56 | 0 |
| ABLIM3 | 0.52 | 0 | RIMKLB | 0.51 | 0 | KCNK2 | 0.54 | 0 |
| SERPINE1 | 0.59 | 0 | CILP | 0.79 | 0 | GLIS3 | 0.51 | 0 |
| BACH2 | 0.59 | 0 | ADAMTS16 | 0.65 | 0 | GLI3 | 0.52 | 0 |
| BMP6 | 0.51 | 0 | LTBP2 | 0.5 | 0 | ABI3BP | 0.53 | 0 |
| OBSL1 | 0.58 | 0 | CRTAC1 | 0.66 | 0 | PNMA8A | 0.52 | 0 |
| CDO1 | 0.62 | 0 | PTPRN | 0.6 | 0 | CHI3L2 | 0.51 | 0 |
| IQSEC3 | 0.52 | 0 | AEBP1 | 0.56 | 0 | EGR3 | 0.52 | 0 |
| ERMN | 0.51 | 0 | RAB3B | 0.51 | 0 | HOXC9 | 0.52 | 0 |
| FNDC1 | 0.62 | 0 | CNR1 | 0.55 | 0 | MFAP5 | 0.58 | 0 |
| AR | 0.53 | 0 | HOXB-AS2 | 0.52 | 0 | PCDHGA10 | 0.57 | 0 |
| THBS2 | 0.62 | 0 | GAS1 | 0.62 | 0 | CHRDL1 | 0.66 | 0 |
| MGP | 0.52 | 0 | VGLL3 | 0.55 | 0 | CARD11 | 0.52 | 0 |
| RASSF9 | 0.57 | 0 | SSC5D | 0.53 | 0 | CCL19 | 0.56 | 0 |
| C4A | 0.51 | 0 | COL8A1 | 0.54 | 0 | HSPB7 | 0.53 | 0 |
| FGF19 | 0.81 | 0 | ALOX12P2 | 0.54 | 0 | CADM3 | 0.55 | 0 |
| GJC2 | 0.53 | 0 | ZBTB7C | 0.52 | 0 | IGHV4-39 | 0.69 | 0 |
| FOSB | 0.64 | 0 | PLXNA4 | 0.53 | 0 | ALPK3 | 0.51 | 0 |
| FAM184A | 0.57 | 0 | KLHL14 | 0.52 | 0 | TNFRSF19 | 0.55 | 0 |
| COL1A1 | 0.5 | 0 | PLPP4 | 0.56 | 0 | CALB2 | 0.58 | 0 |
| COMP | 0.82 | 0 | PCDHB16 | 0.57 | 0 | KIF1A | 0.52 | 0 |
| IGFN1 | 0.61 | 0 | FSIP2 | 0.51 | 0 | SPOCK1 | 0.56 | 0 |
| UCHL1 | 0.53 | 0 | PTGIS | 0.58 | 0 | SOX8 | 0.56 | 0 |
| INHBA | 0.51 | 0 | SLC34A2 | 0.56 | 0 | PRIMA1 | 0.52 | 0 |
| APOD | 0.6 | 0 | PLIN1 | 0.51 | 0 | DSG3 | 0.71 | 0 |
| FGF2 | 0.5 | 0 | FDCSP | 0.69 | 0 | COL11A1 | 0.55 | 0 |
| LRRC4C | 0.54 | 0 | KRT13 | 0.52 | 0 | CNTN1 | 0.54 | 0 |
| DDR2 | 0.51 | 0 | DCLK1 | 0.5 | 0 | KLRG2 | -0.6 | 0 |
| NGFR | 0.55 | 0 | SFRP4 | 0.69 | 0 | SFRP1 | 0.56 | 0 |
| CACNA1E | 0.54 | 0 | NELL2 | 0.54 | 0 | ZFHX4 | 0.53 | 0 |
| HLA-DPB2 | 0.54 | 0 | MEOX2 | 0.56 | 0 | ISM1 | 0.53 | 0 |
| COL10A1 | 0.68 | 0 | SOX2 | 0.66 | 0.01 | SPRR1B | 0.51 | 0.01 |
| HPN | 0.6 | 0 | SLC14A1 | 0.58 | 0.01 | CHRDL2 | 0.52 | 0.01 |
| FAM30A | 0.5 | 0 | TCP11 | 0.53 | 0.01 | IGHD | 0.53 | 0.01 |
| NTRK2 | 0.61 | 0 | CHIT1 | 0.57 | 0.01 | AQP5 | 0.5 | 0.02 |
| SEMA3D | 0.52 | 0 | KRT16 | 0.54 | 0.01 | EGF | -0.52 | 0.02 |
| MXRA5Y | 0.51 | 0 | L1CAM | 0.52 | 0.01 | MUC5AC | 0.59 | 0.02 |
| LONRF2 | 0.5 | 0 | MARCO | 0.59 | 0.01 | ALDH1L1 | 0.52 | 0.02 |
| AMH | 0.5 | 0.01 | IGLV4-60 | 0.58 | 0.01 | IGHV3-7 | 0.51 | 0.02 |
| HAND2 | 0.52 | 0.01 | FREM1 | 0.53 | 0.01 | IGHV1-2 | 0.54 | 0.02 |
| KRT17 | 0.52 | 0.01 | NTSR1 | 0.52 | 0.01 | DEFA6 | -0.66 | 0.02 |
| SCEL | 0.58 | 0.01 | KRT6A | 0.63 | 0.01 | UGT2B17 | 0.6 | 0.04 |
| RIMBP2 | 0.5 | 0.01 | IGF2 | 0.64 | 0.01 | SLC39A2 | -0.52 | 0.05 |
| MYH11 | 0.52 | 0.01 | C6orf15 | 0.62 | 0.01 |  |  |  |

Table S3 Diagnostic value of stemness subtypes predictor

|  | Training set | Testing set | Overall set |
| --- | --- | --- | --- |
| AUC | 0.923 | 0.941 | 0.928 |
| Sensitivity | 0.799 | 0.837 | 0.812 |
| Specificity | 0.886 | 0.918 | 0.891 |
| Cut-off | 0.428 | 0.343 | 0.412 |

Figure S2 Comparison of distribution of classification in our study and previous study [1].


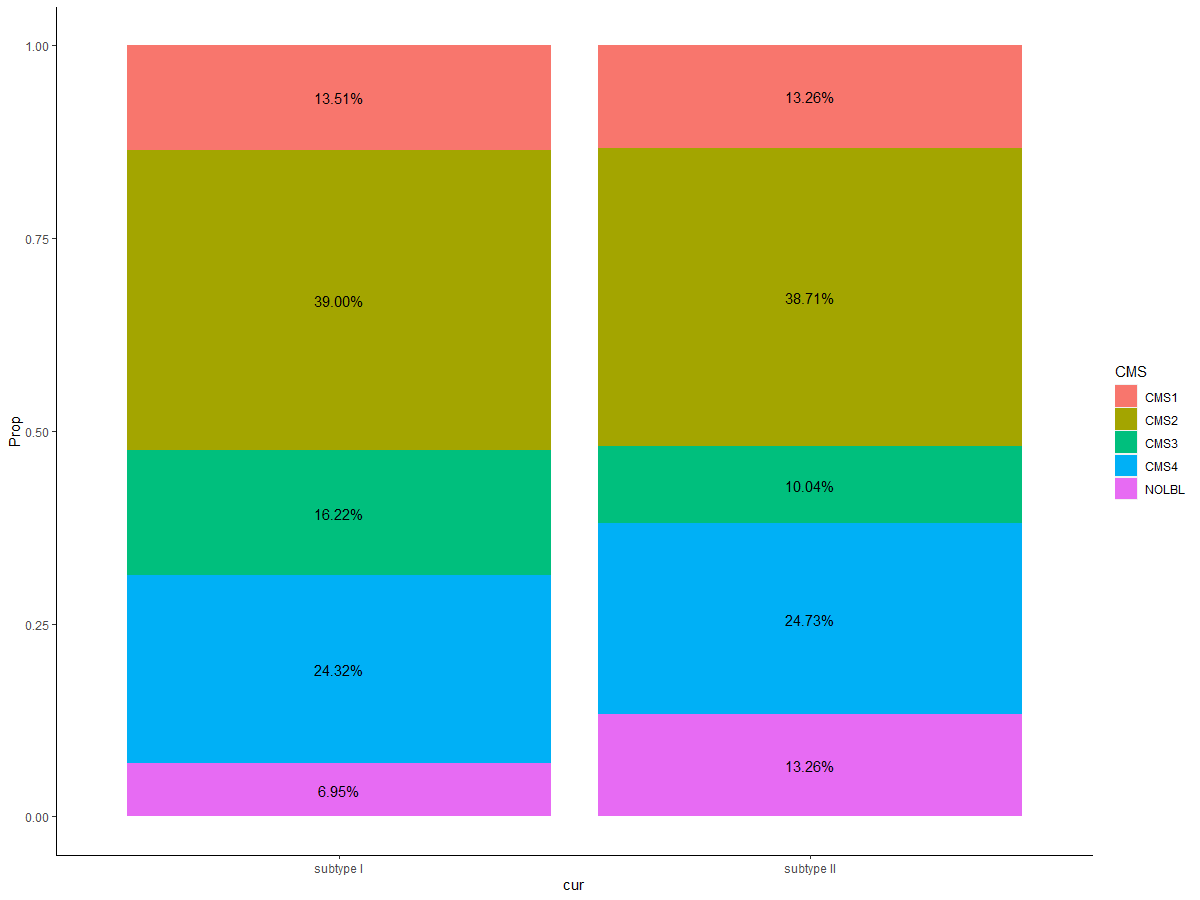


**Reference**

1. Guinney J, Dienstmann R, Wang X, de Reynies A, Schlicker A, Soneson C, et al. The consensus molecular subtypes of colorectal cancer. *Nat Med*, (2015) 21(11), 1350-6 doi:10.1038/nm.3967
